# Metabolic Profile of Sow Blood Serum after Weaning

**DOI:** 10.1155/2022/2372585

**Published:** 2022-05-29

**Authors:** Nadezhda Vladimirovna Bogolyubova, Roman Anatolievich Rykov, Sergei Yurevich Zaitsev

**Affiliations:** Department of Physiology and Biochemistry of farm animals, Federal Research Center for Animal Husbandry named after Academy Member L.K. Ernst, Dubrovitsy 60, Podolsk Municipal District 142132, Moscow Region, Russia

## Abstract

The aim of our research was to determine the content of protein, carbohydrate, lipid, and mineral metabolites, as well as an antioxidant status of the sow's blood after weaning and to calculate the correlation between these parameters. The experiment was carried out on twenty clinically healthy crossbred sows (Yorkshire *× *Landrace). Twenty sows were allocated to one of two groups: (1) 1 day after weaning (group 1, *n* = 10) and (2) 8 days after weaning (group 2, *n* = 10). The basis of the sow diet was SK-1 compound feed, balanced in terms of nutrients and energy in accordance with modern standards and the recommended feeding regimen. Sows blood samples were taken and analyzed for the metabolites of nitrogenous, carbohydrate-lipid, and mineral metabolism and indicators of antioxidant status. The results showed that, in group 2, the total protein content was 89.07 g/l, which is 10.2% higher than that in group 1 (*p* < 0.05); it was mainly achieved due to the globulin fraction. The urea increased by 19.1% (*p* < 0.05), but the concentrations of magnesium and chlorides decreased by 20.2% (*p* < 0.01) and 5.43% (*p* < 0.05), as well as the alkaline phosphatase and ALT activities decreased by 42.5% (*p* < 0.05). Strong positive correlations of the ceruloplasmin with total protein (0.672) and very strong with globulins (0.780) were observed. There was a strong negative correlation between the AST activity and the TBA-AP content, as well as the values of phospholipids and TAWSA. There are moderate negative correlations of the TBA-AP with magnesium, TAWSA and ALT activity, and moderate positive correlations of the TBA-AP with total protein, albumin, triglycerides, and cholesterol. The revealed tendencies and dependencies will serve as the theoretical basis for the development of practical methods for regulating the level of free-radical reactions.

## 1. Introduction

For the timely prevention and elimination of metabolic disorders, as well as to ensure the optimal metabolic status and productive health of farm animals, it is necessary to constantly monitor the state of metabolism and conduct preventive or therapeutic (in cases of clinical manifestation) measures. In this regard, a study of the physiological and biochemical status (PhBS) of the organism of productive animals is relevant from the animal health point of view. Special attention in this matter should be paid to females in the “special” periods of physiological stress (such as pregnancy and lactation).

It is important to highlight that sow health plays a fundamental role in producing healthy offspring [1]; herds with good management and healthy sows produce more litters and piglets per year [2].

It is well-known [3, 4] that the animal breed, in addition to the animal physiological state, also plays an important (sometimes, key) role in the metabolic processes occurring in the body, in particular— sows. For example, Trukhachev V.I. and coauthors [3] found that the body of large white breed pigs reacts significantly to the course of gestation, which is manifested in an increasing activity of the following enzymes: *γ*-glutamyltransferase (GGT, up to 34.42 ± 1.18 U/l, *p* < 0.05), alkaline phosphatase (ALP, up to 82.47 ± 2.51 U/l), alanine aminotransferase (ALT, by 37.9%), and lactate dehydrogenase (LDH). These effects indicated the activation of protein and carbohydrate metabolism, including glycolysis and other major biochemical processes. The authors [3,4] found that, after farrowing, the parameters of the studied enzymes gradually return to the values before gestation. The only exception is the ALT value, which is associated with toxic processes and the effect of fetuses on the pigs' body during pregnancy [3]. Changes in the biochemical parameters of pigs' blood in postnatal ontogenesis have also been studied by other researchers [4].

Along with the study of all physiological and biochemical indicators that characterize the state of metabolism in the body, it is relevant to assess the pro- and antioxidant status of the body, whose indicators are known to be interrelated with animal health, including the state of the immune and reproductive systems [1].

The researchers pay attention to the study of the indicators of lipid peroxidation (LPO) and antioxidant status in the body of pigs of different sex and age groups [5, 6]. Thus, the authors of [5, 6] consider the effect of the use of various forms of biologically active substances (in particular, vitamins [5], natural antioxidants [6], etc.) on the state of the antioxidant system and the intensity of lipid peroxidation reactions in a pigs' body.

In the case of negative environmental influences on productive animals, various dysfunctions occur, and then the coming pathology can cause various damages to pig breeding and animal husbandry [7, 8]. It is noted that, under the influence of unfavorable environmental factors on the animals and especially with the development of various variants of pathology, disorders in the hemostatic system often occur, which negatively affects their physiological status [9].

Shabunin S. V. and coauthors [10] presented the results of studying the dynamics of indicators of pro- and antioxidant status, as well as cytokine profile of piglets under stress caused by weaning and transfer to rearing in an industrial pig breeding complex. The authors of [10] found that 3–10 days after exposure of technological stress to animals, the content of malondialdehyde, medium-molecular peptides, the index of endogenous intoxication, and the level of anti-inflammatory cytokines increased. The results obtained by the authors [10] on the interdependence of pro- and antioxidant status indicators and cytokine profile in the development of oxidative stress associated with various stress factors are a key link to animal adaptation. The completion of the adaptation process of piglets to new conditions was observed at around 20 days after stress exposure. The process of “lipoperoxidation” is a universal nonspecific pathogenetic link in the development of many diseases because of the increasing intensity of free radical processes and high toxicity of the products (formed in the LPO process). According to Maksimov G. V. and coauthors [11], the study of antioxidant protection indicators can serve as an important method for assessing the stress resistance of animals.

Galochkin V. A. and coauthors [12] showed that there is a strong relationship between antioxidant defense systems and natural resistance. Thus, an increase in free radical reactions of lipid peroxidation leads to a violation of the function of antigenic information processing and the synthesis of antibodies. At the same time, a number of immune-modulators block the lipid peroxidation of plasma and subcellular membranes, protecting them from the action of peroxides and free radicals, which are formed especially often in metabolically active cells (macrophages and neutrophils), and thereby preserve the normal structure and function of the membranes [13].

According to Tregubova N.V. and coauthors [14], animals with higher productivity (HP-animals) reacted more strongly to physiological stress, which led to an increased formation of lipid peroxidation products. Due to this, an antioxidant status of such HP-animals was restored more slowly, and the involution processes in the reproductive organs were slowed down, which ultimately contributed to the weakening of the body's resistance [14].

The researchers also concern the study of the state of the antioxidant system in the animal's body at various diseases [15].

The authors are comparing the LPO processes that are occurring in the body of different breed animals. The blood samples of clinically healthy animals of different breeds contain a certain background of free radicals (primary, secondary, and terminal LPO products) and products of oxidative modification of major proteins. The highest level of products of lipid peroxidation and oxidative modification of proteins, as well as a high iron content, was recorded for pigs [16].

According to the authors [17], the deterioration of the reproductive ability of mammals during stressful influences can be caused by oxidative stress and the formation of an excessive amount of reactive oxygen species (ROS) in the body [17]. Oxidative stress can also trigger pregnancy complications [18]. Ovulation is an oxidative process that is known to promote the production of reactive oxygen species in many tissues [19]. Other researchers have also shown that oxidative stress plays a role in the pathophysiology of infertility and promotes infertility of weaning multiparous sows [20, 21].

Other authors compared the indicators of antioxidant protection in the body of “repair pigs” and sows of 2 and 4-5 farrowing. The activity of some antioxidant defense (AOD) enzymes was lower in repair pigs, which indicates that the enzymatic link of AOS is less effective in these animals. This was also consistent with the indicators of oxidative damage. The combined results of studies, including the analysis of systemic markers of oxidative stress, showed that sows of 4-5 farrows more effectively counteract the harmful effects of oxidative stress [22].

Vencova I. Yu. studied the changes of the antioxidant status depending on the physiological state of animals [23]. Usenko S. A. [24] studied the dynamics of LPO indicators of pig's blood depending on the reproductive cycle. According to the authors [24, 25], such changes are observed due to the constantly changing hormonal background, which, by regulating metabolic processes, accelerates or slows down the processes of peroxidation during the reproductive cycle [25]. Lubina E. N. et al. [25] proposed to consider that the period of gestation and lactation in sows was accompanied by the LPO activation. This is evidenced by an increase in the MDA concentration of the animal serum and a decrease in the activity of the particular enzymes (superoxide dismutase, ceruloplasmin, and catalase) which prevent the formation of peroxides or destroy them. The use of biologically active compounds (BAC), vitamins, and minerals in the last third of pregnancy and during lactation in the diet of sows can help to reduce the negative effects of physiological stress by stimulating the body's AOD system. Increased oxidative stress in pregnant sows may be associated with a decrease in the availability of antioxidants during late pregnancy and lactation, which begins to normalize by the end of the lactation period. In this regard, other authors also suggest to increase the content of vitamins E and A in the diet during pregnancy in order to compensate a significant loss of these nutrients [26, 27].

Thus, the study of biochemical processes, including the state of the antioxidant system, is relevant for assessing the general physiological and biochemical status of the body and monitoring the health status of animals of various age and sex and physiological groups. Particular attention here should be paid to the parent herd, on the health of which the quality of the offspring depends.

Given the fact that the metabolic status and indicators of the antioxidant defense of the body affect the health of animals, the state of the immune system, and reproductive function, it is of interest to study these markers at various stages of the reproductive cycle. The obtained data can serve for the accumulation of a database for the development of reference intervals in pigs of different physiological conditions. In addition, there are few data in the literature that characterize the correlation between metabolic parameters and indicators of antioxidant protection. This is important for determining new markers of the state of the antioxidant defense of the body.

Despite the great biological significance, the processes of free radical lipid oxidation and the state of the body's antioxidant defense system during the development of adaptive and pathological processes in pigs, as well as their relationship with blood biochemical parameters, have not yet been sufficiently studied. One of the central issues of the participation of free radical reactions in physiological processes is to clarify the possibility and mechanisms of their regulation. In this regard, it is interesting to study the correlation between blood biochemical parameters and indicators of the state of the body's antioxidant system.

In the studies of modern authors, attention is paid to biochemical indicators in the body of sows, but there is not enough data on the state of the antioxidant system at various stages of the reproductive cycle. We believe that the study of indicators of antioxidant protection in the body of sows, both separately and in conjunction with biochemical markers, will be useful for monitoring the health of animals and developing reference intervals for monogastric animals of different sex, age, and technological groups.

The aim of our research was to determine the content of protein, carbohydrate, lipid, and mineral metabolites, as well as an antioxidant status of the sow's blood (after weaning) and to calculate the correlation between these parameters.

## 2. Materials and Methods

### 2.1. Animals

Sows were maintained at a breeder facility. The experiment was carried out on twenty clinically healthy crossbred sows (Yorkshire* × *Landrace). Twenty sows were allocated to one of two groups: (1) one day after weaning (group 1) and (2) 8 days after weaning (group 2).

The research was conducted in accordance with the requirements of the European Convention for the Protection of Vertebrate Animals used for Experimental and other Scientific Purposes (ETS No. 123, Strasbourg, 1986). The research was approved by the bioethical commission of the L.K. Ernst Federal Research Center for Animal Husbandry (protocol #2021–02/1, dated Feb 01, 2021).

The basis of the diet was SK-1 compound feed, balanced in terms of nutrients and energy in accordance with modern standards and the recommended feeding regimen [28]. The composition of feed SK-1 was as follows: barley 43%, wheat 28%, wheat bran 10.6%, soybean meal 15%, and mineral additives 3.4%. The pigs had constant access to water.

### 2.2. Serum Analysis

Blood collections were carried out mid-morning. Blood samples were collected from the jugular vein from each sow on the same day. An 18 g needle was used to collect blood samples into 2 × 8 ml VACUETTE® serum tubes with blood clotting activator (Greiner Bio-One, Austria) that were centrifuged within 4 h of collection at 5000 g for 5 min. Samples were sent to the laboratory (the Department of physiology and biochemistry of farm animals at the Federal Research Center for Animal Husbandry named after Academy Member L.K. Ernst) and analyzed on an automatic biochemical analyzer ChemWell (Awareness Technology, USA) using reagents from Analyticon Biotechnologies AG (Germany), Spinreact (Spain) and Deacon (Russia).

Methods used: total protein—by the biuret method (9104), albumin—by the colorimetric method (9136), globulins—by calculation, A/G—by calculation, urea—according to Berthelot (1001329), creatinine—by the Jaffe kinetic method (448), alanine aminotransferase (ALT)—by the UV kinetic (1187), aspartate aminotransferase (AST)—by the UV kinetic (1177), alkaline phosphatase (ALP)—by the UV kinetic (1625), glucose—by the enzymatic-glucose oxidase (4341), Triglycerides—by the enzymatic-colorimetric method (41031), phospholipids—by the enzymatic-colorimetric method (1001140), total bilirubin—by the Walters and Gerarde method (804), cholesterol—by the enzymatic-colorimetric method (41021), calcium—by the O-cresolphthalein complexone method (10100), phosphorus—by the colorimetric method (1914), and magnesium—by the colorimetric method (1001280).

### 2.3. Lipid Peroxidation (LPO) Assay

Lipid peroxidation (LPO) level in serum samples was measured by the standard method (reaction with the thiobarbituric acid) using kits “Agat-Med” (Russia). The values of the thiobarbituric acid active products (TBA-AP) were expressed in *μ*mol⁄ml. The activity of ceruloplasmin (CP) was measured by the method of Revin [29].

The total amount of water-soluble antioxidants (TAWSA) was measured by the amperometric method using the device “TsvetYauza-01-AA” (“Khimavtomatika”, Russia). The TAWSA values were determined by measuring the strength of the electric current arising during the oxidation of molecules on the surface of the working electrode at a potential of ∼500 mV. TAWSA was measured equivalent to gallic acid as in [30]. For this, the “working solutions” were prepared from a gallic acid solution (100 mg/dm3) for calibration with a mass concentration of 0.2, 0.5, 1.0, and 4.0 mg/dm^3^. An amount of 2.2 mmol/dm^3^ phosphoric acid solution was used as an “eluent.” The results of measuring the total antioxidant activity of the samples were statistically processed using the MS Excel program.

The ratio of TBA-AP to CP was calculated by the authors.

### 2.4. Statistical Analyses

The quantity data are presented as mean value (*M*) and standard error (±SEM). Pearson correlation test was used to determine relationship between the obtained biochemical parameters. All the data were analyzed by using the software packages “Microsoft Office Excel 2003” and “R Studio” (version 1.1.453) (https://rstudio.com). The results of the statistical analysis were considered significant at *p* < 0.05.

## 3. Results

### 3.1. Biochemistry

As the first results of the conducted biochemical studies, some differences in the studied parameters between two groups of animals were established (Table 1). Thus, in the group of sows 8 days after weaning, the total protein content was 89.07 g/*l*, which is 10.2% higher than in the other group of sows (*p* < 0.05). This was mainly achieved due to the globulin fraction (45.21 g/l in group 2 versus 39.11 g/l in group 1) at *p* < 0.05. With an increase in the number of days after weaning, the body of sows also increases the urea content by 19.1% at *p* < 0.05. The content of metabolites of carbohydrate-lipid metabolism—cholesterol and triglycerides—was also higher in the group of sows 8 days after weaning, compared with the animals of the other group.

As for the metabolites of mineral metabolism, in our studies, we noted a decrease in the concentration of magnesium by 20.2% (*p* < 0.01), chlorides by 5.43% (*p* < 0.05), and the activity of alkaline phosphatase by 42.5% (*p* < 0.05) in sow's group 2 as compared to the first one.

### 3.2. Parameters of LPO and AOD

The parameters of LPO and AOD in the body of sows are presented in Table 2. The content of ceruloplasmin in the body of sows significantly increased (by 36.3% at *p* < 0.01), as well as the amount of TBA-active products, by the lengthening of the period after weaning. At the same time, the content of water-soluble antioxidants in animal's blood increased only slightly (by 6.4%) (Table 2). The conjugacy of the processes of lipoperoxidation and antioxidant protection can be estimated by calculating the ratio of a number of components of these systems. The ratio of the TBA-AP content to the CP level in group 2 of sows was slightly lower (by 5.9%) than those of group 1 (Table 2), probably due to the relatively high activity of ceruloplasmin. This shows that after weaning, the antioxidant system of the sow body (as a whole) responds adequately to changes in the intensity of lipid peroxidation processes.

### 3.3. Correlations

After conducting a correlation analysis between the studied parameters, the moderate and strong positive correlations were observed between the serum concentrations of ceruloplasmin and total protein (0.672) or globulin (0.780) (Table 3). A moderate negative correlation was observed between the activity of aspartate aminotransferase (AST) and the content of TBA-active products, as well as the concentration of phospholipids and TAWSA (Table 3). Weak negative correlations are observed between the concentration of TBA-AP in the blood and the content of magnesium and TAWSA, as well as ALT activity (Table 3), and weak positive correlations are observed between TBA-AP and the content of total protein, albumins, triglycerides, and cholesterol (Table 3).

We found a weak negative correlation between the serum content of ceruloplasmin and magnesium and a positive correlation of the same degree between the content of ceruloplasmin and urea, ceruloplasmin, and cholesterol.

## 4. Discussion

Thus, during lactation and after weaning of piglets, the biochemical processes of sow's body are changing significantly. It is well known [32, 33] that protein metabolites (total protein and its fractions and urea) and lipid metabolites (cholesterol and triglycerides) are the most important components for the milk synthesis. An increase of these protein and lipid metabolites in the blood serum of sows (8 days after weaning of piglets) indicates that these precursors of the secret of the breast are less consumed after the cessation of milk formation in the animal body. This is associated with an increase in their concentrations in the body's metabolic “pool.”

Authors of [33] studied the effect of the genotype and the time after farrowing on the indicators of metabolic processes in the body of sows. For example [33], in the first month of lactation (in comparison with the third) in the body of sows, there are reduced levels of total protein and its fractions and increased concentrations of lipid metabolites, in particular, cholesterol. This proves that lactation has a strong effect on the metabolism of sows during the first weeks after farrowing since the piglets “need for nutrients” is very high at the beginning of lactation.

These data are proved by authors of [34] who studied the biochemical blood parameters of sows and determined the reference intervals for all biochemical parameters.

Ceruloplasmin is currently considered as one of the main antioxidants of blood plasma [28]. In addition to the transport of copper, and it neutralizes O2-radicals like superoxide dismutase and binds Fe^2+^ and Cu^2+^ ions, removing them from the fenton reaction, which is one of the key processes in the initiation of LPO [35]. In our studies, the values of this indicator for the experimental groups were 113 and 154 mg/l, respectively, with a significant difference between the groups (*p* < 0.01). Thus, the concentration of ceruloplasmin, which plays an important role in the antioxidant defense of the body, significantly increases in sow's blood after weaning. It is known that healthy sows (after weaning of piglets) are characterized by optimal lipid composition of red blood cells and low activity of lipid peroxidation in them [36]. Our data (Tables 1–3) differ from the results obtained by other scientists [37], who observed higher values of this indicator in the blood of weaned piglets as compared to adult pigs [37]. Probably, such a high CP activity is a response to the increased LPO processes under the influence of stress factors on the piglet's body.

Analyzing the nature of changes of such an indicator as TBA-AP, it is necessary to take into account its priority value in comparison with other LPO indicators since the composition of TBA-AP includes a number of highly reactive compounds that act on all cell structures. According to the modern data, these compounds provide a multifactorial phenomenon, which is defined as endogenous intoxication [38]. In our study (Table 2), the concentration of TBA-AP was at the level of 1.92 mmol/l for the group 1 (of sows immediately after weaning) and at the level of 2.48 mmol/l for the group 2 (of sows at 8 days after weaning). Such values are consistent with the data of other authors, according to which this indicator is in the range of 2.63–4.81 mmol/l [39].

Thus, after the termination of lactation in sows, the state of the LPO and AOP systems also changes. This is expressed in an increase of a ceruloplasmin content in the blood after weaning of piglets, which indicates an increase in the level of AOP after the termination of lactation. A small decrease in the ratio of TBA-AP to CP in sow's group 2 due to the high activity of ceruloplasmin (Table 3) shows that the antioxidant system of the sow body (as a whole, after weaning) reacts more adequately to the changes in the intensity of lipid peroxidation processes.

Berchieri-Ronchi C. B. and coauthors [18] showed that sows (exposed to increased systemic oxidative stress throughout the gestational and lactation periods) did not fully recover before the weaning period [18]. The authors [18] studying the indicators of antioxidant protection during the entire period of gestation and lactation and affecting 5 days after weaning found the following: (a) a DNA damage significantly increased in the second quarter of the gestational period; (b) it was maintaining increased damage throughout the lactation period; (c) it still did not fully recover during the weaning period.

We observed moderate positive correlations between the content of ceruloplasmin and total protein and strong positive between ceruloplasmin and globulins (Table 3). This is because ceruloplasmin, as a highly valuable copper-containing protein, presents in blood plasma at high activity. A moderate negative correlation was observed between the serum phospholipid content and TAWSA (Table 3). Water-soluble antioxidants include those that perform their protective function in the cytosol of cells, intercellular fluid, blood plasma, lymph-ascorbic acid, citric acid, nicotinic acid, sulfur-containing compounds (cysteine, homocysteine, lipoic acid, and benzoic acid), phenolic compounds, and flavonoids. This, apparently, explains the inverse relationship of their content (Table 3) with phospholipids.

Interesting in our opinion are the correlations between the content of magnesium in the blood and the concentration of TBA-AP. It is known that magnesium participates in the stabilization of cell membranes, acting as a membrane- and “cytoprotective” factor. It is known that the composition of TBA-AP includes a number of highly reactive compounds that act on all cell components, including DNA, and lead to disorganization of the cell membrane structure [38]. It is obvious that, with a decrease in the content of such a metabolite of mineral metabolism as magnesium, the level of lipid peroxidation products in the blood increases slightly.

## 5. Conclusions

The revealed tendencies and dependences will serve as the theoretical basis for the development of practical methods for regulating the level of free-radical reactions (within the biological capabilities of the sow's body at different physiological conditions), which is of great practical importance.

The authors are conducting further studies of genetic and phenotypic factors that affect the blood parameters of sows and the state of the antioxidant defense system in different periods of the reproductive cycle. In this context, it would be important to estimate the correlation between the content of some other biochemical parameters in the sow's blood (in particular, enzymes and trace elements that are part of some enzymes of the antioxidant system), for the perspective of managing the processes of adaptation and increasing the resistance of animals, stimulating growth and development, and increasing their productivity.

## Figures and Tables

**Table 1:** Indicators of metabolic processes in the body of sows after weaning.

Parameters	Groups			Norm [31]
	1 (*n* = 10)	2 (*n* = 10)	*P* value	
Total protein, *g*/l	80.84 ± 3.12	89.07 ± 1.79^*∗*^	0.95	63–90
Albumins, *g*/l	41.73 ± 1.36	43.86 ± 1.24	0.80	28–46
Globulins, *g*/l	39.11 ± 1.92	45.21 ± 1.58^*∗*^	0.80	26–56
A/ G	1.07 ± 0.03	0.98 ± 0.05	0,40	0.5–1.7
Total bilirubin, *μ*M/l	8.74 ± 1.16	7.32 ± 1.44	0,60	—
Urea, mmol/l	7.86 ± 0.53	9.36 ± 0.32^*∗*^	0.98	2.6–7.6
Glucose, mmol/l	3.25 ± 0.38	2.79 ± 0.27	0.70	—
Cholesterol, mmol/l	2.07 ± 0.16	2.48 ± 0.12^∗∗^	0.995	—
Calcium, mmol/l	2.61 ± 0.05	2.73 ± 0.06	0.90	1.9–3.2
Phosphorus, mmol/l	3.03 ± 0.16	3.33 ± 0.19	0.80	1.8–4.8
Magnesium, mmol/l	0.99 ± 0.03	0.79 ± 0.04^∗∗^	0.995	0.8–1.7
Iron, mmol/l	25.63 ± 3.19	27.38 ± 1.84	0.50	15–39
Chlorides, mmol/l	114.0 ± 1.73	107.81 ± 2.04^*∗*^	0.98	—
ALT, IU/l	51.57 ± 5.17	43.93 ± 5.77	0.40	21–97
AST, IU/l	51.53 ± 5.05	43.75 ± 2.67	0.70	14–72
Alkaline phosphatase, IU/l	113.35 ± 16.67	65.15 ± 6.38^*∗*^	0.98	43–226
Creatinine, *μ*M/l	158.53 ± 8.40	140.0 ± 14.97	0.90	81–200
Triglycerides, mmol/l	0.90 ± 0.03	1.00 ± 0.03^∗∗^	0.99	—
Phospholipids, mmol/l	2.20 ± 0.09	2.06 ± 0.09	0.70	—

The obtained differences compared to the control are statistically significant at *p*: ^*∗*^*p* < 0.05, ^*∗∗*^*p* < 0.01, and ^*∗*∗∗^*p* < 0.001. A/ G: albumin to globulin ratio; ALT: alanine aminotransferase; AST: aspartate aminotransferase.

**Table 2:** Indicators of lipid peroxidation and antioxidant protection in the body of sows after weaning.

Parameters	Groups		*P* value
	1(*n* = 10)	2 (*n* = 10)	
TBA-AP, *μ*mol/l	1.92 ± 0.21	2.48 ± 0.22	0.90
Ceruloplasmin, mg/l	113 ± 10.0	154 ± 8.7^∗∗^	0.995
TAWSA, mg/l	17.86 ± 0.55	19.00 ± 0.73	0.80
TBA-AP/CP	0.017	0.016	

The obtained differences compared to the control are statistically significant at ^*∗∗*^*p* < 0.01. TBA-AP: thiobarbituric acid active products; TAWSA: total amount of water-soluble antioxidants; TBA-AP/CP: ratio of TBA-AP to ceruloplasmin.

**Table 3:** Correlation between indicators of metabolic processes and indicators of lipid peroxidation and antioxidant protection in sows.

Parameters	Group of animals (*n* = 10)		
	TAWSA	TBA-AP	Ceruloplasmin
Total protein	−0.225	0.305	0.672^*∗*^
Albumin	−0.209	0.348	0.207
Globulin	0.101	0.205	0.780^∗∗^
Urea	0.117	0.274	0.489
Triglycerides	0.231	0.475	0.095
Phospholipids	−0.546	0.140	−0.151
Cholesterol	−0.182	0.456	0.410
ALT	0.075	−0.307	−0.292
AST	−0.044	−0.527	−0.114
Magnesium	−0.153	−0.350	−0.476
TAWSA	1.0	−0.316	0.089
TBA-AP, µmol/L	−0.316	1.00	−0.089
Ceruloplasmin	0.089	0.089	1.00

Correlations: 0.9–1.0 = very strong; 0.70–0.90 = strong; 0.50–0.70 = moderate; 0.30–0.50 = weak; 0–30 = very weak. The obtained differences compared to the control are statistically significant at ^*∗*^*p* < 0.05 and ^∗∗^*p* < 0.01. Abbreviations: ALT: alanine aminotransferase; AST: aspartate aminotransferase; TBA-AP: thiobarbituric acid active products; TAWSA: total amount of water-soluble antioxidants.

## Abbreviations

ALP: Alkaline phosphatase
ALT: Alanine aminotransferase
AOD: Antioxidant defense
AST: Aspartate aminotransferase
BAC: Biologically active compounds
CP: Ceruloplasmin
LPO: Lipid peroxidation
TAWSA: Total amount of water-soluble antioxidants
TBA-AP: Products that react with thiobarbituric acid.
